# Could Urology’s Antimicrobial Stewardship Be Enhanced by the Routine Use of the Meares and Stamey Test?

**DOI:** 10.3390/diagnostics15081002

**Published:** 2025-04-14

**Authors:** Simone Botti, Tommaso Ceccato, Michele Rizzo, Giovanni Liguori, Alessandro Zucchi, Alessandro Palmieri, Truls E. Bjerklund Johansen, Tommaso Cai

**Affiliations:** 1 Urology Division, Santa Chiara Regional and Teaching Hospital, Provincial Health Care Agency (APSS), 38123 Trento, Italy; simone.botti@apss.tn.it (S.B.); tommaso.ceccato@apss.tn.it (T.C.); 2 Department of Urology, University of Trieste, 34121 Trieste, Italy; mik.rizzo@gmail.com (M.R.); gliguori@units.it (G.L.); 3 Department of Urology, University of Pisa, 56121 Pisa, Italy; zucchi.urologia@gmail.com; 4 Department of Urology, University Federico II, 80013 Naples, Italy; info@alessandropalmieri.it; 5 Institute of Clinical Medicine, University of Oslo, 0025 Oslo, Norway; t.e.b.johansen@medisin.uio.no; 6 Department of Urology, Oslo University Hospital, 0025 Oslo, Norway; 7 Institute of Clinical Medicine, University of Aarhus, 8210 Aarhus, Denmark; 8 Centre for Medical Sciences (CISMed), University of Trento, 38122 Trento, Italy

**Keywords:** Meares and Stamey test, antimicrobial stewardship, prostatitis, chronic bacterial prostatitis, antibiotic resistance

## Abstract

**Background/Objectives**: Chronic bacterial prostatitis (CBP) is a prevalent urological condition significantly impacting patients’ quality of life. Accurate diagnosis is essential to differentiate bacterial from non-bacterial prostatitis and to guide appropriate antimicrobial therapy. In the context of antimicrobial resistance (AMR), the Meares and Stamey (M&S) test is a valuable diagnostic tool for targeted antibiotic use and a valuable antimicrobial stewardship (AMS) measure. Despite its clinical relevance, its adoption is limited by practical and logistical challenges. **Methods**: Relevant databases were searched by using methods recommended by the Preferred Reporting Items for Systematic Reviews and Meta-Analysis guidelines. The keywords used included “Meares and Stamey test,” “antimicrobial stewardship and prostatitis,” and “chronic bacterial prostatitis and Meares.” **Results**: We enclosed seven studies: one single-center prospective observational comparative study, two national surveys, three cross-sectional studies, and one consensus conference. The M&S test remains the gold standard for diagnosing CBP, offering high specificity in identifying bacterial infections localized within the prostate. The test enables precise pathogen identification and facilitates targeted antimicrobial therapy. Despite its clinical relevance, its adoption is hindered by procedural complexity, patient discomfort, and the apparent need for specialized personnel and facilities. Alternative diagnostic methods, such as the two-glass pre- and post-massage test (PPMT), have demonstrated comparable diagnostic sensitivity while being more practical and time-efficient. Additionally, emerging microbiological techniques are under investigation to increase the M&S test’s sensitivity. **Conclusions**: The M&S test plays a crucial role in AMS by ensuring targeted antimicrobial therapy in CBP. Overcoming its limitations through patient stratification, clinician education, and the integration of emerging microbiological techniques is essential to enhance its applicability in modern urological practice.

## 1. Introduction

Urogenital tract infections in men comprise urinary tract infections (UTIs) and male accessory gland infections (MAGIs). The high prevalence and long treatment duration of up to 3 months lead to a widespread use of antibiotics. Therefore, the application of antimicrobial stewardship (AMS) principles is essential for optimizing patient outcomes and minimizing the societal impact of antimicrobial resistance (AMR) [1]. Among MAGIs, prostatitis is the most prevalent condition with a high impact on men’s quality of life. Prostatitis frequently leads patients to seek consultation in urology clinics. Nevertheless, prostatitis syndrome has always been a nosological challenge as regards antibiotic treatment. We still refer to the classification given in 1995 by the National Institutes of Health-sponsored consensus conference [2], that identified four prostatitis categories: I Acute bacterial prostatitis, II Chronic bacterial prostatitis (CBP), III A Inflammatory chronic non-bacterial prostatitis—CPPS (this was known as “nonbacterial prostatitis”), III B Non-inflammatory CPPS (this was known as “prostatodynia”), and IV Asymptomatic inflammatory prostatitis (histological prostatitis) [3]. The first two categories comprise approximately 10% of prostatitis cases and are the only types thought to have a microbiological etiology [4]. The remaining 90% of cases consist of non-bacterial syndromes, which pose great challenges in both diagnosis and treatment [1,2,3]. It has long been established that in non-bacterial forms of prostatitis, antimicrobial agents are neither justified nor effective [5,6]. In these patients, microbiological analysis of midstream urine cultures and samples obtained from the Meares and Stamey test (M&S) remains an essential tool for accurate diagnostic and etiological assessment of bacterial prostatitis and avoidance of unnecessary antibiotics [3], particularly considering the role of bacterial biofilm [7]. Due to side effects for patients and the risk of collateral effects on the society, the time when doctors prescribed long term courses with broad spectrum antibiotics based on symptoms only, is long overdue. In men with symptoms of urogenital infection, we need to know if the prostate is involved, and if so, also the identity and resistance of the pathogen. This review aims to provide a comprehensive analysis of the role of the Meares and Stamey test in the diagnosis of bacterial prostatitis and the potential of this test as a contemporary antimicrobial stewardship measure. To reach this aim, we put forth two research queries: (i) Should the Meares and Stamey test be regarded as a must before the prescription of antibiotics to a man with prostatitis-like symptoms in order to improve patient management and reduce unnecessary use of antibiotics? (ii) Will a wider use of the Meares and Stamey test improve antimicrobial stewardship in urological practice?

## 2. Materials and Methods

To respond to the defined research questions, we performed a systematic review of all available studies providing evidence for the clinical and microbiological benefits and limitations of the Meares and Stamey test and its role as an antimicrobial stewardship measure in urological practice.

### 2.1. Literature Search

The PubMed database, Cochrane CENTRAL, Web of Science, and Scopus were searched for publications. Every reference listed in pertinent publications was also examined and evaluated. The following keywords were included in the search strategy: “Meares Stamey” AND “prostatitis”/OR “bacterial prostatitis”/OR “chronic prostatitis.” The filters we employed were clinical studies, humans, English language, and adult. The following PICO questions were formulated: Patients (adult patients with prostatitis-like symptoms), Intervention (diagnostic pathway by using the Meares and Stamey test), Control (diagnostic pathway by using other laboratory methods), and Outcomes (diagnostic accuracy of the Meares and Stamey test and its role in antimicrobial stewardship in urology).

### 2.2. Inclusion and Exclusion Criteria

Inclusion criteria were manuscripts in English, addressing in adult males (>18 years) the role of the Meares and Stamey test in the diagnosis of bacterial prostatitis and the potential of this test as a contemporary antimicrobial stewardship measure. No limitations pertaining to the study’s nature have been considered.

### 2.3. Literature Review

Titles and abstracts were used to screen for initial study inclusion. When abstracts were not enough to establish if the study satisfied inclusion or exclusion criteria, a full-text review was conducted. Two independent reviewers (S.B. and T.Cec.) conducted the review. A supervisor (T. Cai) was consulted to sort out any disagreements between the two reviewers. The study was performed in line with the Preferred Reporting Items for Systematic Reviews and Meta-Analyses (PRISMA), the recommendations of the European Association of Urology Guidelines office for conducting systematic reviews and meta-analyses, and previous studies [8,9]. The systematic review was conducted using all of the included studies. Reviews that have already been published on the subject have been used to provide context and to advance certain aspects of the field that the original research did not address.

### 2.4. Analysis, Presentation, and Quality and Risk of Bias Assessment

Because of the low number of records, study findings were synthesized into a narrative report in four sections. To assess the quality and risk of bias, we used the ROBINS-I tool [10]. Two independent trialists performed this analysis under the supervision of a senior trialist.

## 3. Results

**Figure 1:**
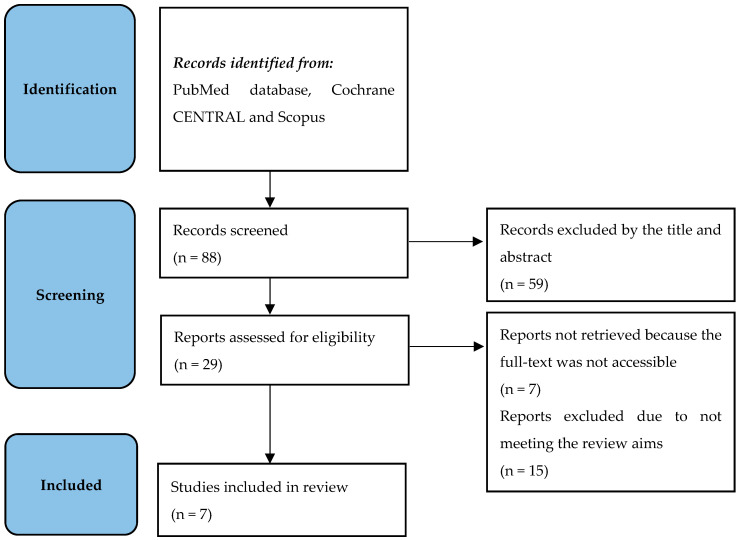
The flowchart of the literature search in line with the PRISMA statement.

The structured literature research identified a total of 88 unique records. Twenty-nine were retrieved and screened for relevance. Seven papers underwent final review (Figure 1).

### 3.1. Evidence Synthesis

#### 3.1.1. Antimicrobial Resistance in Urology and Antimicrobial Stewardship

The therapeutic advantages of antibiotics in treating bacterial urogenital infections are well-documented [9]. Their excessive and inappropriate use has, however, played a major role in the rise of antimicrobial resistance among uropathogenic bacteria, which now represents a significant global public health concern [11]. The World Health Organization has officially recognized AMR as one of the most pressing healthcare challenges of the 21st century [12]. The increasing prevalence of multidrug-resistant bacterial strains has made the treatment of infections, including those affecting the urinary tract and prostate, increasingly difficult, necessitating urgent and effective intervention strategies. In response to this growing threat, antimicrobial stewardship programs have been progressively developed since the 1990s [13]. These initiatives are designed to improve patient outcomes, ensure the rational and cost-effective use of antibiotics, and minimize the unintended adverse consequences of antimicrobial therapy. Uncontrolled and inappropriate antibiotic prescribing has, in fact, contributed not only to AMR development but also to healthcare-associated infections, such as those caused by *Clostridioides difficile*, drug toxicity, and the selection of highly virulent bacterial strains [13].

To achieve effective AMS implementation in urological practice, we adopt a multifaceted strategy that integrates various approaches aimed at optimizing antibiotic use and preventing resistance. Continuous education and training of healthcare professionals are crucial. Educational programs should focus on pharmacokinetic and pharmacodynamic principles, equipping clinicians with the necessary knowledge to select the most appropriate antibiotic, optimize dosage and duration of therapy, and assess potential adverse effects [14,15]. Moreover, strict adherence to evidence-based clinical guidelines is a key component of AMS, as it facilitates informed antibiotic selection, discouraging the routine empirical use of broad-spectrum agents unless they are absolutely necessary [3]. Implementing regular audits and reviews of antibiotic prescribing patterns within urological departments is another essential step. By analyzing trends in antibiotic use, healthcare providers can identify patterns of overuse or misuse, refine their prescribing behaviors, and adopt targeted strategies to ensure judicious antimicrobial application [14]. The establishment of multidisciplinary AMS teams is another critical component of AMS programs. These teams should include urologists, infectious disease specialists, microbiologists, pharmacists, and infection control practitioners, all working collaboratively to develop and oversee antimicrobial policies, monitor resistance trends, and guide clinical decision-making in complex cases. This interdisciplinary approach ensures that antimicrobial therapy is evidence-based, appropriately targeted, and continuously monitored for effectiveness [14].

Additionally, a crucial aspect of effective AMS is the regular reassessment of antibiotic therapy, particularly within 48–72 h after initiation. As microbiological test results become available, clinicians should re-evaluate treatment efficacy and determine whether de-escalation to a narrower-spectrum antibiotic or discontinuation of therapy is warranted. This adaptive strategy minimizes unnecessary exposure to antibiotics while ensuring that patients receive the most targeted and effective treatment for their specific condition [15]. Ultimately, the successful implementation of AMS in urology requires a comprehensive, collaborative, and evidence-based approach that integrates education, adherence to clinical guidelines, continuous monitoring, and multidisciplinary expertise. By incorporating these strategies into routine clinical practice, urologists and other healthcare professionals can play a pivotal role in combating antimicrobial resistance, preserving the efficacy of existing antibiotics, and improving overall patient care [14,15].

#### 3.1.2. Diagnostic Benefits and Limitations of the Meares and Stamey Test

Originally described in 1968, the Meares and Stamey 4-glass test (M&S) remains an essential diagnostic tool for chronic bacterial prostatitis [3,15]. The technique involves the systematic collection and microbiological analysis of four distinct urine and prostatic secretion samples to localize infections within the lower urinary tract accurately. The process includes obtaining (I) first-voided urine (VB1), which provides information on urethral flora; (II) midstream urine (VB2), reflecting the microbial composition of bladder urine; (III) expressed prostatic secretion (EPS), collected through prostatic massage and (IV) post-massage voided urine (VB3), which helps identify prostatic bacteria dislodged during the procedure. By comparing bacterial concentrations across these specimens, the test facilitates the distinction between bacterial prostatitis and other prostatic or pelvic conditions, with a good profile of accuracy (overall sensitivity of 75% and overall specificity of 95%) [16,17]. In some series, Zegarra Montes et al. [18] and Cai et al. [19] found up to 81% of positive Meares and Stamey tests in patients stratified for clinical presentation and referred to dedicated centers, highlighting the subset of individuals who may benefit from precisely targeted antimicrobial therapy. Over the years, a simplified, more cost-effective, and less time-consuming 2-glass pre-massage and post-massage test (PPMT) has been developed, demonstrating comparable diagnostic sensitivity, in particular when EPS is not obtained [12,20,21]. In general, both tests demonstrate high specificity but limited sensitivity, indicating that a negative result does not entirely exclude the presence of chronic bacterial prostatitis. Unfortunately, this aspect, combined with a common belief among urologists that the M&S test is not a routine microbiological examination, has limited its practical application and continued to produce ambiguous results, which further contributes to its infrequent use [19,22,23]. Consequently, the Meares and Stamey test is either not performed or replaced by semen culture, which, according to clinical guidelines, should not be routinely utilized alone in the diagnosis of CBP because of its high prevalence of false positives due to contamination problems [3,24,25].

#### 3.1.3. The Meares and Stamey Test as an Antimicrobial Stewardship Measure

As a result of the previously stated findings, the Meares and Stamey test plays a pivotal role in identifying chronic prostatitis as a bacterial condition, thereby guiding the selection of appropriate antibiotic therapy. In this context, the test is essential within the framework of the 5-D model of antibiotic stewardship, where establishing an accurate diagnosis (the first D) represents the step with the greatest potential impact on optimizing antimicrobial use [12]. Although CBP significantly impacts quality of life, it is not a life-threatening condition. Consequently, immediate initiation of treatment is not always required; therefore, there is sufficient time to provide symptomatic management for the patient while investigating the etiology of the symptoms. A precise identification of the underlying cause is essential as several factors contribute to the symptoms of CBP and make it a challenging disease to treat and prevent complications during the treatment period.

The prostate epithelium is not easy to reach for a drug that must possess lipid solubility, low serum protein binding, and appropriate molecular properties, including size, shape, and ionization [26,27]. This aspect influences the number of antibiotics we can use and the time needed to eradicate a microorganism (at least 4–6 weeks of treatment for non-intracellular bacteria) [3,12,24]. Another challenge is given by the bacteria involved in CBP. Among these, the Enterobacteriaceae, especially *Escherichia coli*, are the most prevalent, but other frequently found microorganisms are Enterococci, Staphylococcus, *Pseudomonas aeruginosa*, and the genital mycoplasmas and atypical bacteria (*Chlamydia trachomatis*, *Mycoplasma genitalium*, *Mycoplasma hominis*, *Ureaplasma parvum* and *Ureaplasma urealyticum*) [24,28,29]. The fungal cause should be considered only in immunocompromised patients. Many of these bacterial strains are established to be able to produce organized aggregation encased in a self-generated extracellular matrix called biofilms. These structures, different from the “planktonic” form, enhance microbial survival by increasing resistance to antimicrobial agents and immune defenses, playing a key role in chronic infections, persistent symptoms, and recurrent infections [29,30,31,32]. Another way for bacteria to survive is the development of enzymes or the evolution of mechanisms of resistance to withstand the effects of antimicrobial agents, rendering treatments less effective [33,34,35]. Moreover, antibacterial agents appear to be less effective when targeting only the so-called causative bacterium compared to addressing the entire bacterial spectrum often identified in the biological sample, potentially through a combination of antibiotics [27]. The treatment of chronic bacterial prostatitis is, therefore, inherently complex due to both the overlap of symptoms with non-bacterial prostatitis and the intrinsic characteristics of the prostate and its causative pathogens. For the above reasons, treatment regimens are often long-lasting and involve broad-spectrum antibiotics. If this treatment is not justified, it significantly violates the principles of antimicrobial stewardship. The Meares and Stamey test facilitates targeted treatment and helps us reduce overtreatment. It is, therefore, a potent AMS measure in urology.

#### 3.1.4. Challenges and Limitations of the Meares and Stamey Test

The M&S test serves as a fundamental diagnostic tool to identify and manage chronic CBP. Despite its ability to localize infection within the prostate, the widespread implementation of the M&S test in routine practice is hindered by multiple challenges. One limitation is patient discomfort during the procedure due to the prostatic massage, which some individuals may find uncomfortable or invasive. However, the M&S test may eliminate the need for a urethral swab and lessen the overall discomfort that patients experience from this procedure when used in the diagnostic pathway of suspected sexually transmitted infections.

Additionally, the procedure is time-consuming, requiring careful stepwise collection and microbiological analysis of four distinct urine and prostatic fluid samples. These requirements place additional burdens on both clinicians and healthcare facilities, as the test demands a dedicated clinical setting, as well as trained urologists. Furthermore, although the M&S test is known for its high specificity, it suffers from limited sensitivity, meaning that a negative result does not necessarily exclude the presence of CBP [19]. This limitation raises concerns about false-negative results, potentially leading to missed diagnoses and inappropriate treatment choices. Alternative approaches such as PPMT have been developed to overcome these challenges, maintaining comparable diagnostic accuracy [3,21]. Additionally, patient stratification based on symptomatology and referral of patients to dedicated centers can improve test sensibility [19].

Another strategy for overcoming barriers to M&S test implementation is to enhance the education of clinicians, particularly urologists and general practitioners. Misconceptions regarding the test’s utility, feasibility, and diagnostic accuracy contribute to its underutilization. Incorporating training programs into medical education and continuing professional development initiatives can help improve awareness and increase adoption in clinical settings.

Moreover, emerging microbiological techniques, such as polymerase chain reaction and next-generation sequencing, have expanded the understanding of the microbial spectrum involved in CBP—particularly in culture-negative cases—but their practical clinical utility remains uncertain [36,37]. These techniques can detect bacterial DNA even in the absence of viable bacteria, making the interpretation of results and adherence to AMS principles challenging. Additional research is required to determine how these advanced methods can be integrated into current diagnostic workflows and explore whether they can complement traditional culture-based techniques. Future studies focusing on immune profiling and cytokine analysis may also provide novel insights into the inflammatory pathways underlying CBP and further refine diagnostic and therapeutic strategies [37]. In summary, while the M&S test remains a cornerstone in the diagnosis of CBP and in antimicrobial stewardship, its practical application is limited by several challenges that should be addressed deliberately. A combination of simplified diagnostic procedures (PPMT), improved patient selection, and enhanced clinician education can help mitigate these limitations. Since other available microbiological techniques are still under investigation, the M&S test remains the only validated method for the diagnostic assessment of CBP.

Table 1 summarizes all selected and included studies.

Table 2 shows the Risk of bias ROBINS-I of the included studies.

## 4. Conclusions and Future Perspectives

The M&S test is an essential tool in the accurate diagnosis and management of chronic bacterial prostatitis. By enabling precise pathogen identification, this diagnostic method contributes significantly to targeted and appropriate antibiotic use. Although we did not find any studies demonstrating a reduction in the unnecessary use of broad-spectrum antibiotics in urogenital infections by using the M&S test, we consider the opportunity for a culture-based antibiotic treatment to be a strong argument for a wider use of the M&S test in MAGIs. The AMR crisis calls for measures to facilitate the prudent use of antibiotics. We will, therefore, argue that (i) the M&S test should be regarded as a must before the prescription of antibiotics to a man with prostatitis-like symptoms, and (ii) a wider use of the Meares and Stamey test is an important means to improve antimicrobial stewardship in urological practice.

We believe the high diagnostic specificity of the M&S test outweighs procedural complexity and patient discomfort; the adoption of simplified alternatives, such as the PPMT, alongside the training of clinicians and better patient selection may enhance the practical application of the principles of Meares and Stamey. Further research into emerging microbiological techniques and their integration with traditional methods is crucial in refining diagnostic accuracy and optimizing treatment strategies and timely antimicrobial stewardship efforts in urological practice.

## Figures and Tables

**Table 1:** The table shows the summary of all clinical trials included in the review.

Author	Year	Type of Study	Aim	Findings Description
Zegarra Montes LZ, et al. [18]	2008	Cross-sectional study	To assess the diagnostic accuracy of semen and urine culture in the diagnosis of chronic bacterial prostatitis.	The Meares and Stamey test remains important for the diagnosis of chronic bacterial prostatitis in everyday clinical practice.
Cai T, et al. [19]	2024	Cross-sectional study	To analyze if the diagnostic yield of the Meares and Stamey test could be improved by a pre-test categorization of patients due to undergo a Meares and Stamey test.	Symptom-based patient selection and dedicated staff members will increase the diagnostic yield of the Meares and Stamey test and reduce the number of unnecessary tests.
Nickel JC, et al. [21]	2006	Cross-sectional study	To investigate the diagnostic accuracy of the 2-glass pre-massage and post-massage test, in comparison with the Meares–Stamey 4-glass test, to detect inflammation and bacteria in men with chronic prostatitis/chronic pelvic pain syndrome.	The pre-massage and post-massage test has strong concordance with the 4-glass test and is a reasonable alternative when expressed prostatic secretions are not obtained.
McNaughton Collins M, et al. [22]	2000	National survey	To describe the results of a national mail survey of practicing urologists in 1998 to examine the diagnosis and treatment of chronic prostatitis.	Urologists frequently diagnose chronic prostatitis but rarely perform the four-glass diagnostic test. Use of the four-glass test does not appear to affect urologists’ antibiotic treatment patterns.
de la Rosette JJ, et al. [23]	2000	National survey	To describe the results of a national mail survey among urologists in the diagnosis and treatment of chronic prostatitis.	The first choice of therapy in prostatitis patients is antibiotics without performing Meares–Stamey test.
Magri V, et al. [24]	2019	Consensus conference on prostatitis	To describe the results of a consensus conference and expert opinion meeting on prostatitis.	The Meares and Stamey test remains important for the diagnosis of chronic bacterial prostatitis in everyday clinical practice.
Kogan MI, et al. [27]	2022	Single-centre prospective observational comparative study	To investigate if the antimicrobial therapy targeted on Meares and Stamey test is more effective than standard therapy in chronic bacterial prostatitis.	Antimicrobial therapy, according to the Meares–Stamey test is a more effective alternative to standard therapeutic regimens for chronic bacterial prostatitis.

**Table 2:** The table shows the risk of bias of all studies included in the review.

Authors	Type of Bias	Bias Grading	Overall Bias
Zegarra Montes LZ, et al. [18]	Bias in the selection of participants in the study	Moderate risk	Moderate risk
Cai T, et al. [19]	Bias in the selection of participants in the study	Moderate risk	Moderate risk
Nickel JC, et al. [21]	None	None	None
McNaughton Collins M, et al. [22]	Not applicable	Not applicable	Not applicable
de la Rosette JJ, et al. [23]	Not applicable	Not applicable	Not applicable
Magri V, et al. [24]	Not applicable	Not applicable	Not applicable
Kogan MI, et al. [27]	Bias due to confounding Bias in the selection of participants in the study	Moderate risk Moderate risk	Moderate risk

## Data Availability

Not applicable.

## Abbreviations

The following abbreviations are used in this manuscript:

UTIs	Urinary Tract Infections
AMS	Antimicrobial Stewardship
AMR	Antimicrobial Resistance
CBP	Chronic Bacterial Prostatitis
M&S	Meares and Stamey test
VB1	First-voided urine
VB2	Midstream urine
EPS	Expressed Prostatic Secretion
VB3	Post-massage voided urine
PPMT	Pre-massage and Post-massage test
